# Caesarean Delivery Influences Breast Milk Composition—A Narrative Review

**DOI:** 10.3390/nu18020207

**Published:** 2026-01-09

**Authors:** Maciej Maj, Joanna Robaczyńska, Maja Owe-Larsson, Hubert Rytel, Bożena Kociszewska-Najman, Jacek Malejczyk, Izabela Róża Janiuk

**Affiliations:** 1 Department of Histology and Embryology, Center of Biostructure Research, Medical University of Warsaw, 02-091 Warsaw, Poland; 2 Clinic of Neonatology and Rare Diseases, Medical University of Warsaw, 02-091 Warsaw, Poland; 3 Institute of Health Sciences, Faculty of Medical and Health Sciences, University of Siedlce, 08-110 Siedlce, Poland

**Keywords:** breastfeeding, caesarean section, CS, human milk composition, infant health, lactation

## Abstract

Delivery by caesarean section (CS) is increasingly common worldwide and has been associated with altered health outcomes in offspring, which can be partially mitigated with breastfeeding. Interestingly, the mode of delivery itself may influence the composition of human milk. The aim of this narrative review was to comprehensively examine current evidence on the impact of CS on breast milk composition and to discuss its potential implications for neonatal and infant health. A literature search of the MEDLINE database was conducted in July 2025. It identified 1212 studies addressing associations between mode of delivery and human milk components, of which 54 were included in the qualitative synthesis. Available evidence suggests that CS is associated with transient, lactation stage-dependent alterations in breast milk composition, most pronounced in colostrum and transitional milk. Reported changes include differences in macronutrients, mineral content, immune-related molecules, hormones, antioxidants, microbiota, microRNA profiles, and other bioactive components. Findings related to mature milk are less consistent and often influenced by confounding factors. While some CS-associated alterations may slightly reduce the beneficial effect of breastfeeding, e.g., reducing certain antimicrobial or nutritional components, other changes seem to be potentially advantageous for the neonate/infant after CS, in particular in immune-related factors. Overall, the clinical significance of these compositional differences remains unclear, as no studies have directly linked CS-related changes in milk composition to long-term infant outcomes. Further well-designed longitudinal studies are needed to clarify these associations. Regardless of delivery mode, breastfeeding remains the optimal feeding strategy and a key intervention to support infant health after CS.

## 1. Introduction

### 1.1. Trends and Health Implications of Caesarean Delivery and Breastfeeding

Caesarean section (CS), also known as c-section or surgical delivery, is an increasingly common method of childbirth [1]. While in 1990 about 7% of all births were surgical, in 2018 this rate reached 21.1%, and is predicted to rise to 29% by 2030 [2]. The prevalence of CS varies by region, being highest in the Americas (42.8% in Latin America and the Caribbean) and lowest in Sub-Saharan Africa (5%) and in Melanesia, Micronesia, and Polynesia (3.6%), with marked differences also observed between neighboring or closely located countries; for example, CS accounts for the majority of births in Poland, whereas the rate in Czechia is approximately half as high, and in France CS represents only 19.3% of all births [2,3]. Although CS is generally considered safe, its high and rising rate raises concerns regarding public health impact [1], especially given that the World Health Organization (WHO) recommends an optimal CS rate of 10–15%, noting that higher rates do not further reduce maternal or neonatal mortality [4]. The increase in CS rates is multifactorial and is often associated with maternal choice rather than strict medical indications [1]. Elective CS is perceived as predictable, convenient, and pain-free [1,5,6], and may also be favored by physicians for financial reasons [5]. However, factors such as advanced maternal age [7], obesity [8], and even symptomatic COVID-19 infection during pregnancy [9] may also have contributed, to some extent, to this upward trend in recent years. Despite certain advantages, such as lower risk of urinary incontinence, genital prolapse, and, e.g., faster involution of the uterine cavity [10,11], CS remains a surgical procedure with higher risks of infection, longer hospitalization [10,12], and complications in subsequent pregnancies, including uterine rupture, abnormal placentation, and ectopic pregnancy [13].

CS may also affect the offspring’s health in both short- and long-term manners [13]. CS has been linked to allergic diseases—including food allergies, asthma, atopic dermatitis [14]; chronic inflammatory and autoimmune diseases—including celiac disease, Crohn’s disease, rheumatoid arthritis [15,16]; autism spectrum disorder [17]; and metabolic diseases—type II diabetes, or obesity [18]. These outcomes are attributed to altered physical, hormonal, and microbial exposures during birth, including disrupted gut colonization due to the lack of contact with maternal vaginal microbiota [13,19].

Breastfeeding can mitigate these adverse effects, since maternal milk provides protection against infections [20], atopy [21,22,23], autoimmune diseases [24], and childhood obesity [25]. It is not only the optimal source of macronutrients, minerals, and vitamins for the growing child [26], but also a rich source of immunomodulatory components such as lactoferrin, antibodies, oligosaccharides, cytokines, immune cells, and beneficial bacteria, especially colostrum—the first milk produced up to around day 5 postpartum [27,28]. These components support the gut microbiota and immune system development, protecting against the listed conditions.

Because of the beneficial effects of human milk on the offspring’s health and development, the WHO and UNICEF recommend initiating exclusive breastfeeding within the first hour postpartum and maintaining it for at least 6 months [29].

### 1.2. Effect of Caesarean Delivery on Breastfeeding Initiation Rate, Frequency, and Milk Volume

Women after CS tend to face more challenges with breastfeeding compared to mothers after vaginal delivery. Numerous studies have shown that CS, especially planned CS, delays the onset of breastfeeding and is associated with its shorter duration [30]. Moreover, the volume of breast milk produced after CS is also diminished in comparison to the vaginal route delivery, although these differences seem to disappear by day six postpartum [31]. Additionally, women after CS, especially elective CS, tend to breastfeed less frequently [32]. While these short-term effects may not directly impact child development, they seem to increase the risk of early breastfeeding cessation [31,32].

Several factors are contributory, mainly related to the surgical procedure and postoperative care, though medical indications for CS also play a minor role [33]. During vaginal delivery, oxytocin and prolactin—hormones essential for lactation—are released in a pulsatile pattern. The disruption of endocrine secretion accompanying CS is perceived as one of the main causes of delayed initiation of breastfeeding after CS. Postpartum oxytocin levels are lower in mothers after elective CS. Moreover, hormonal profiling during early breastfeeding demonstrates that women who deliver vaginally exhibit more oxytocin peaks and higher prolactin concentrations than those who undergo CS [33,34]. In addition, the physiological endocrine response driving lactation may be further impaired in mothers after CS due to delayed early skin-to-skin contact, which is a critical stimulus for the release of lactation hormones [35].

Other barriers include postoperative pain, limited mobility, fatigue, surgical complications, and difficulty positioning for feeding [36,37]. In addition, newborns delivered by CS may exhibit weaker attachment and suction reflexes due to maternal analgesia [38,39] and may experience hormonal imbalances affecting appetite regulation and suckling behavior [33]. These factors discourage mothers after CS from breastfeeding exclusively and may reduce milk supply due to insufficient nipple stimulation [40].

There is also limited evidence showing that planned CS are associated with a higher likelihood of choosing not to initiate breastfeeding and a lower likelihood of seeking lactation support compared with emergency CS or vaginal delivery [41]. In another study, women who had undergone repeated CS were less likely to initiate breastfeeding at all than those who had a vaginal birth after CS, even after adjustment of sociodemographic and medical factors [42]. Although these findings require further investigation, they indicate that the mode of delivery may influence a mother’s decision to start breastfeeding. This may be related to factors such as previous breastfeeding experiences in multiparous women or lifestyle considerations, e.g., the desire to return to work [41].

Although not all of the factors associated with CS can be eliminated, professional support, education, and pro-breastfeeding interventions—especially those promoting early skin-to-skin contact—can significantly improve exclusive breastfeeding rates following CS [37,43].

### 1.3. Aim of the Review

Since CS is a surgical procedure associated with certain anaesthesia schemes, a higher risk of bleeding, longer hospitalization, infections, as well as a neurohormonal imbalance associated with the absence of natural labor [33,44], it might also affect the composition of human milk.

Changes in breast milk composition following CS have been reported, affecting, e.g., macronutrient [45], mineral [46], hormonal [47], antioxidant [48], microbial [49], miRNA [50], and immune-related components of human milk [51]. These alternations are predominantly found in colostrum and transient milk; however, many articles show that mature milk composition changes [52,53].

As emerging evidence suggests that CS can alter human milk composition, this review summarizes the current state of knowledge and considers how these changes may affect offspring health. To our knowledge, this article is the first to provide a comprehensive analysis of the available literature on this topic.

## 2. Search Strategy

A search of the available literature on the correlation between mode of delivery and breast milk composition was performed in October 2025. The MEDLINE database was used to perform this narrative review. The literature search strategy was based on keywords found in previous reviews concerning human milk and CS. Boolean operators (“OR”, “AND”) were used to combine search terms listed below.

The search string consisted of two conceptual domains:

- CS domain: (cesarean OR c-section OR caesarean OR surgical delivery)
- human milk domain: (colostrum OR milk OR breastmilk)

**Figure 1:**
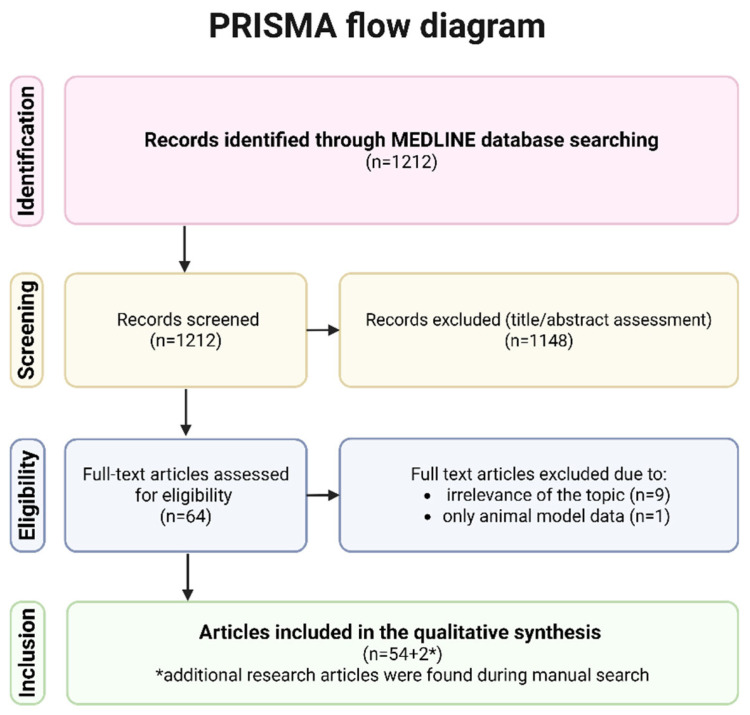
Flowchart of the literature search strategy. Created with BioRender.com.

To ensure broad coverage of the literature, the search strategy aimed to capture as many studies as possible addressing the link between delivery mode and milk composition. Articles were included if they contained relevant data and were based on the analysis of human breast milk, excluding those involving animal samples. Figure 1 illustrates the search strategy applied in this article. Supplementary Materials contain a table summarizing all included articles, including author, year, country, milk collection period, aim of the study, and relevant results.

## 3. Changes in Milk Composition Due to Caesarean Delivery

### 3.1. Nutrients (Other than Minerals)

The available data on the effect of CS on human milk nutrient composition are inconsistent and generally indicate small, lactation stage-dependent alterations rather than major differences.

Colostrum samples collected on the second day postpartum showed significantly lower total protein levels in the CS group compared with vaginal delivery, while energy content and fat and carbohydrate concentrations did not differ between groups [54]. A similar study based on colostrum obtained at the same time point reported comparable findings [45].

A prospective longitudinal study analysing milk samples collected on days 3, 7, and 15 postpartum found that colostrum from preterm infant mothers after vaginal delivery (on day 3) contained higher fat concentrations than colostrum from CS mothers [55]. However, other macronutrients, including protein and carbohydrate concentrations, as well as fat concentrations on days 7 and 15, did not differ significantly between the CS and vaginal group. Authors noted that the lack of significance might be due to the relatively small sample size, and that the trends toward higher fat (days 7 and 15) and carbohydrate concentrations in the vaginal delivery group might reach significance in a larger cohort [55]. The results of this study in terms of fat concentrations are in line with a different article, where colostrum of mothers (of infants born in term) following CS collected 72 h postpartum had significantly lower levels of triglycerides compared to colostrum obtained from mothers after vaginal delivery. This study also found that the CS group had higher levels of several phosphatidylinositol and phosphatidylserine classes [56].

The dynamic nature of macronutrient changes during early lactation may partially explain discrepancies between findings from samples collected on postpartum days 2 and 3. Although dynamic and transient, the colostrum macronutrient composition appears to be slightly less favourable for neonates born via CS; however, it is unlikely to have a major impact on offspring health. These rapid changes in colostrum composition could also explain the lack of significant correlation between the mode of delivery and total lipid, lactose, or protein content reported in another study, where milk samples were collected within a 0–3-day postpartum interval [57].

Data regarding mature milk is less consistent, likely affected by multiple factors beyond mode of delivery. A retrospective cohort study revealed that breast milk collected from mothers following vaginal delivery (around 15 days postpartum) had higher amounts of lactose compared to breast milk from mothers after CS. However, total protein concentrations in mature milk collected 15 days postpartum did not vary between the groups [58]. Another analysis of samples collected within three months postpartum (without a specific collection day, limiting interpretability) suggested that CS may be associated with higher fat and lower carbohydrate concentrations, while protein levels remained similar [59]. Conversely, a Finnish cohort study reported significantly higher protein concentrations in mature milk from CS mothers (collected 2–3 months postpartum) compared with vaginally delivering mothers [60]. Yet another study, which examined milk at various postpartum intervals (17 ± 3, 30 ± 3, 60 ± 5, 90 ± 5, and 120 ± 5 days), found no significant relationship between mode of delivery and total lipid, lactose, or protein content [57].

Apart from macronutrient content, several studies—mostly examining mature milk—have investigated how the mode of delivery influences the protein profile, fatty acid composition, and metabolomic characteristics of breast milk. An analysis of milk collected one month postpartum found no meaningful differences in multiple nutrients, including fatty acids, sugars, triacylglycerols, vitamins, and amino acids [61]. In another study, mature milk collected from mothers six weeks after CS, but interestingly not colostrum (collected 0–5 days postpartum), was found to contain lower concentrations of κ-casein, anti-infectious protein—lactoferrin, and β-casein compared with mothers who delivered vaginally [62].

CS delivery was also associated with changes in the breast milk fatty acid composition. A one-month postpartum milk analysis revealed a correlation between CS and lower concentrations of beneficial omega-3 fatty acid docosahexaenoic acid [61]. Another analysis (samples were collected at random time points) showed that the breast milk of mothers who delivered via the vaginal route contained significantly higher anti-inflammatory *n*-3 PUFA, stearic, and palmitoleic acid concentrations, and significantly lower inflammatory fatty acid content and *n*-6/*n*-3 ratio compared to women after CS [63]. An increase in human milk *n*-6/*n*-3 FA ratio may contribute to higher childhood obesity risk [64]. Comparable results were observed in a multicenter European study, which analysed samples of colostrum and mature milk in six different time ranges postpartum. However, when co-variates were adjusted, including infant birthweight, parity, gestational age, and the mother’s origin, the results were no longer statistically significant [57].

A metabolic analysis of human milk one month postpartum revealed additional observations in terms of milk nutrient changes after CS. Vaginal delivery was associated with higher levels of the ketone body and energy metabolite 3-hydroxybutyrate, as well as the oligosaccharide lacto-N-fucopentaose III [65], which are considered beneficial for the infant’s neurodevelopment [66], exerting immunomodulatory effects [65], and shaping the gut microbiome [67]. In contrast, milk from CS mothers contained higher relative abundances of butyrate (a short-chain fatty acid), ethanolamine (a phospholipid precursor), proline, and urea [65], which do not seem to be as advantageous for the infant as, e.g., higher levels of lacto-N-fucopentaose III. Although this study was based on a relatively small sample size, it revealed that differences in milk metabolome composition between CS and vaginal deliveries depended on the country of sample origin, which could be attributed to a wide range of variables, from different delivery protocols to lifestyle and diet [65].

Based on the available data, milk composition changes following CS seem to be transient and could be affected by several other factors, including postpartum day, gestational age, ethnicity, and different intra-operative and postoperative practices in different countries during/after CS [65]. This could be the reason for the observed discrepancies between available data. Overall, a slightly less beneficial nutrient composition of human milk cannot be excluded, especially in terms of colostrum.

### 3.2. Minerals

The mode of delivery seems to be associated with differences in breast milk mineral concentrations, especially trace mineral content in the early days postpartum. Among the minerals studied, the strongest evidence concerns iodine. Colostrum and transitional milk, but not mature milk, of CS mothers contained higher iodine concentrations compared to milk obtained from mothers after vaginal delivery [46,68].

Iodine is an essential milk component. Its deficiency impairs thyroid hormone synthesis, which could affect the physical, neurological, and intellectual development of the offspring [69]. Excessive iodine ingestion could potentially disrupt thyroid function, though, leading to neonatal hypothyroidism or hyperthyrotropinemia as a result of the Wolff-Chaikoff effect. However, a high amount of iodine in the colostrum was observed in a relatively short period; therefore, it is possible that this does not have a significant impact on the offspring [46].

Higher, transient iodine levels in mothers who underwent CS could be a result of povidone-iodine antiseptic use, which can be absorbed into the bloodstream [46,70]. In addition, the surgery itself and anaesthesia have been suggested to affect iodine-containing thyroxin levels, which could potentially be higher in the breast milk of CS mothers, affecting its total iodine content [68]. Nevertheless, it is too early to conclude whether CS has a direct impact on thyroxine levels, due to an insufficient amount of relative data.

Analysis of colostrum collected 3–5 days postpartum showed higher concentrations of iron, copper, zinc, and manganese in Chinese women who delivered vaginally. After adjusting for multiple confounders—maternal characteristics, socioeconomic factors, lifestyle, and infant anthropometrics—only copper remained significantly lower in the CS group, suggesting that CS may reduce copper levels, an essential trace element for infant growth and development [71]. In contrast, a multicenter European study did not confirm lower copper concentrations after CS. Although it initially reported higher manganese, phosphorus, and zinc levels in the vaginal-delivery group and higher iodine and sodium concentrations in the CS group (in the early stages of lactation), these differences became statistically non-significant after accounting for factors such as gestational age, maternal origin, and parity [57].

In summary, more data is required to assess the impact of CS on mineral concentration in human milk. Iodine levels in colostrum seem to be increased in colostrum collected from mothers after CS, whereas in terms of certain minerals—e.g., copper, the available data are not fully consistent. Minerals such as manganese and zinc have been reported at higher concentrations in colostrum from mothers who delivered vaginally, which could be beneficial for the infant. However, these differences did not remain statistically significant after adjustment for covariates [57,71].

It is worth noting that factors such as older maternal age, overweight/obesity before gestation, greater financial level, and smoking habits correlate with a higher probability of CS [72]. Therefore, the observed lower levels of zinc and manganese in colostrum after CS may not be directly caused by the mode of delivery but rather can be explained by factors that correlate with CS, such as maternal age and specific lifestyle.

### 3.3. Immune-Related Molecules and Growth Factors

The mode of delivery may also influence the concentration of growth factors and immunological components—molecules that contribute to the anti-allergic properties of human milk [73]. This is especially important in neonates and infants born via CS delivery due to the higher allergy risk associated with this mode of delivery [14].

In colostrum from mothers after CS (but not in mature milk), higher concentrations of TGF-β2 have been reported [51,74]. Studies also report a similar association for increased TGF-β1, but they are not fully consistent [74,75]. These cytokines support, among others, the development of immune tolerance and the production of IgA in the neonate and infant [51]. CS delivery was also found to be associated with higher colostrum concentrations of mucin 1—an anti-infectious agent and modulator of TGF-β1, T cell response, and toll-like receptor signalling [75].

It is worth noting that there are also studies in which TGF-β levels did not differ significantly between colostrum samples obtained from mothers after CS/vaginal delivery [76]. Compared to the previously mentioned studies investigating TGF-β concentrations in colostrum, this one had a relatively small sample size and examined general non-specific TGF-β concentrations, which could explain the observed discrepancies between data.

Interestingly, TGF-β1 and TGF-β2 concentrations in transient and mature milk did not differ significantly between the CS and vaginal delivery groups, which suggests that the alterations in TGF-β1 and TGF-β2 milk levels after CS, although probably beneficial for the child, take place mainly in the early days postpartum [77].

Colostrum levels of another member of the TGF-β family—activin A, which plays an immunomodulatory role by increasing, e.g., cytokine production and regulating T cell development, did not differ between the CS and vaginal delivery groups [78]. Among other examined growth factors—EGF, IGF-1, and NGF—IGF-1 concentrations in colostrum may be negatively affected by CS, although current evidence is weak and not all studies prove it [76,79]. IGF-1 stimulates intestinal maturation and possibly may be absorbed into circulation [80]. Elevated IGF-1 levels in the colostrum of mothers after vaginal delivery could be beneficial for the neonate’s development. However, more research is needed to confirm these results.

Notably, IGF-1 level disturbances are probably transient in the early postpartum period, since concentrations of IGF-1 (along with adiponectin, leptin, and the IGF-1 metabolite—cyclic Glycine-Proline, which were also measured in this study) in human milk 2–3 months postpartum did not show significant differences between different modes of delivery [60].

Among the analysed IL-6, IL-8, IL-10, IL-13, and TNF, higher concentrations of IL-6 and TNF in transitional and mature milk were found to be independently associated with CS. However, it should be underlined that the analysed probe was relatively low and focused on preterm deliveries [81]. A similar study, based on human milk obtained 7 days and 1 month postpartum, reported significantly higher monocyte chemotactic protein 1 (MCP-1) levels after CS in transient milk (on the 7th day postpartum). Surprisingly, it did not find significant changes in TNF levels [82]. Nevertheless, a separate study reported significantly higher colostrum TNF concentrations [83].

Higher concentrations of pro-inflammatory cytokines may be beneficial at the beginning of lactation, when the neonatal gut is more immature, as they could contribute to an enhanced mucosal defence and support the development of the newborn’s immune system [82]. This may be particularly important in CS neonates who often exhibit an imbalance in gut microbiota [84].

Furthermore, IgA concentrations in colostrum collected on day 2–6 postpartum were higher in the group of mothers that gave birth via CS delivery [74]. Interestingly, in colostrum collected 2–3 days postpartum, mothers who had CS after labor showed higher IgA levels (but not IgG or IgM) compared with vaginal deliveries, whereas elective CS did not show significant differences in IgA, IgG, or IgM concentrations [85]. Differences in sampling time could explain the discrepancies observed between those two studies regarding the effect of elective CS on IgA colostrum concentrations.

Although IgA concentrations may vary depending on the delivery mode, they do not seem to affect mature milk composition. Analysis of mature milk revealed no differences in the concentrations of secretory IgA, IgM, or IgG between different modes of delivery [77,86]. However, milk derived from mothers after CS contained a 2.6-fold higher free secretory component (SC) compared to mothers who delivered their infant vaginally. Although SC is mainly known as a glycoprotein that attaches to IgA, forming a dimeric, secretory IgA form, SC can also neutralize pathogens and their toxins independently. Authors hypothesize that differences in SC concentrations could result from maternal microbiota alterations, induced by CS, influencing mammary epithelial cells to produce more SC in CS mothers [86]. Therefore, higher IgA and SC ingestion by CS neonates and infants could be beneficial, as it strengthens their protection against pathogens.

Colostrum from mothers who delivered by CS has been associated not only with an imbalance in free fatty acid esters of hydroxy fatty acids (FAHFAs) and TAG estolides (two fatty acids and one FAHFA esterified to glycerol)—lipid compounds with anti-inflammatory properties [56]—but also with alterations in antimicrobial components. Specifically, a study focusing on the antimicrobial molecules human β-defensin-1 and β-defensin-2 reported significantly higher concentrations of these proteins in colostrum, but not in mature milk, from mothers after vaginal delivery compared with those after CS [87]. Together with evidence of lower lactoferrin concentrations in mature milk (but not in colostrum) following CS delivery [62], these findings suggest that breast milk after CS may exhibit a modestly reduced anti-inflammatory and antimicrobial defence capacity.

All of these findings indicate that CS may influence the immunological and growth factor composition of human milk, especially in colostrum and transitional milk. The effects on neonates and infants remain unclear and need further investigation, as some changes—such as increased levels of TGF-β1, TGF-β2, and IgA—could be beneficial, whereas reductions in β-defensin-1 and β-defensin-2 might have opposing effects.

### 3.4. Hormones

The concentration of maternal hormones in the breast milk may also depend on the type of delivery.

The colostrum milk levels of β-endorphin, an opioid peptide, on the 4th postpartum day were higher in mothers who delivered vaginally than in mothers who underwent CS [88]. These results are in line with another study of the same author, where β-endorphin levels on the fourth postpartum day were significantly higher in the colostrum of mothers who delivered vaginally, both at term and preterm, than in the colostrum of mothers after CS However, no significant differences in β-endorphin concentrations were observed in mature milk on the 30th day of lactation between delivery modes, and β-endorphin levels declined over time regardless of the mode of delivery. It has been suggested that labor-related pain may contribute to elevated colostrum β-endorphin levels, potentially supporting neonatal adaptation and stress responses following vaginal delivery [47]. Further investigation is needed to determine whether lower β-endorphin levels in milk have any negative effects on the offspring.

Colostrum levels of melatonin—an indoleamine hormone whose presence in breast milk is believed to contribute to sleep-wake cycle synchronization, neurodevelopment, and immune system maturation—especially in preterm infants [89]—appear to differ between women who delivered vaginally and those who underwent CS, in a manner similar to β-endorphin. Specifically, melatonin levels were shown to be lower after CS [90].

Consistently, in another study, nocturnal colostrum melatonin levels were the highest after vaginal delivery, lower after elective CS, and the lowest after an emergency CS [91]. CS possibly affects the rhythm of melatonin secretion. The melatonin concentration in the colostrum of mothers who delivered vaginally was higher at night than during the day. However, no differences were observed in the melatonin secretion during day and night in the colostrum of mothers after CS [83]. This phenomenon may be associated with TNF activity, as the colostrum of women after CS contained higher levels of TNF than that of women who gave birth in a vaginal delivery. Moreover, TNF and melatonin levels were correlated inversely [83]. Thus, the authors suggested that the TNF increase after CS may suppress nocturnal melatonin production [83]. Interestingly, a different study revealed that the levels of melatonin in colostrum samples obtained during daytime are higher in mothers after CS than in mothers who delivered vaginally [92]. However, in contrast to previous studies, the rhythmicity of melatonin secretion with its higher concentrations at night was still present both after CS and vaginal delivery, without significant differences in nocturnal concentration levels between different modes of delivery [92].

Overall, the available evidence, although not fully consistent, possibly due to different study designs—e.g., inclusion/exclusion of preterm deliveries, inclusion of emergency CS, different surgical and anaesthesia protocols among regions [92]—suggests that, similarly to β-endorphin [88], CS probably affects milk melatonin secretion rhythm in the early postpartum days. Further research is needed to fully understand its impact and the potential consequences for neonatal development.

### 3.5. Antioxidants

The balance between antioxidant and pro-oxidant factors in the colostrum may also vary depending on delivery mode. After birth, neonates experience a sharp increase in oxygen exposure and free radical generation compared to the intrauterine environment, resulting in elevated oxidative stress—OS [93].

Studies indicate that OS levels are higher in the colostrum of women who delivered via CS than in those who delivered vaginally [94,95]. Interestingly, antioxidant markers—such as thiol groups, vitamin C, ferric reducing ability, or catalase—also appear elevated after CS [48,95]. However, another study found that the total antioxidant status was reduced in CS colostrum [94]. Therefore, although certain antioxidants may be elevated in the colostrum of women who delivered via CS [48], the overall antioxidant capacity may still be reduced due to increased oxidative stress and a more pronounced reduction in other antioxidants. Moreover, differences in geographical, clinical, and nutritional contexts may also contribute to the observed variability between studies and cannot be excluded. No differences in antioxidant capacity have been found in transient and mature milk of mothers after vaginal and CS delivery [82].

Higher oxidative stress in the colostrum after CS could be explained by surgery and anaesthesia [95]. However, many other factors, including environmental and maternal influences, may also contribute to this phenomenon. Therefore, this difference cannot be assigned just to the delivery mode [96].

### 3.6. Microbiota

The microbiome is a dynamic structure that varies among individuals and changes in response to different environmental factors [97]. The microbiota has various functions in the human body. It plays a protective role against pathogens, helps extract energy and nutrients from food, and shapes the immune system [98]. An appropriate microbiota composition is particularly crucial for neonates and infants born via CS, who often have disrupted bacterial colonisation. This is because neonates born via the vaginal route acquire bacteria from maternal vaginal and perianal microbiota. In contrast, those delivered by CS predominantly acquire microbes from the maternal skin and the surrounding environment [99].

Breast milk is a reservoir of a diverse range of bacterial genera, including *Firmicutes*, *Proteobacteria*, *Actinobacteria*, *Streptococcus*, and *Staphylococcus* predominantly [100]. Studies have shown that several bacterial species are transferred via maternal milk from the mother to the neonate’s/infant’s gut, including, e.g., Bifidobacterium species. This is why breastfeeding could alleviate the negative effects of CS on offspring microbiota composition [101]. Interestingly, the mode of delivery may contribute to differences in microbiota content, as many studies have reported higher microbial diversity and a higher prevalence of probiotic bacteria, e.g., *Lactobacillus* spp., after vaginal delivery [49]. The available data are not fully consistent, though, and indicate that some results, such as those concerning *Bifidobacterium* and *Lactobacillus* spp., did not reach statistical significance. However, this could also be due to, e.g., small sample sizes [102].

One study reported lower microbial diversity and an altered microbial composition in the milk of CS mothers one month postpartum, which could contribute to a higher allergy risk, as atopic children are known to have a reduced gut microbial diversity [103]. Another study also found skewed microbial composition after CS. Interestingly, these changes seemed to have a long-term effect, as they were present in colostrum and were still present in breast milk at 1 and 6 months postpartum. Additionally, the microbiota profiles in samples from women after non-elective CS were more similar to those from the vaginal delivery group than from women who underwent elective CS, which could be due to the presence of physiological stress or hormonal signals [104].

Colostrum from CS mothers has also been associated with a higher abundance of environmental bacteria, belonging to the, e.g., *Pseudomonas* spp., *Staphylococcus* spp., and *Prevotella* spp. genera [105]. A three-month postpartum breast milk analysis revealed a higher *Firmicutes*-to-*Bacteroidetes* ratio, considered a marker of gut dysbiosis, and an increased abundance of opportunistic and pathogenic bacteria in the CS group compared with the vaginal delivery group [52]. Another study observed small alterations in bacterial composition between unscheduled CS and vaginal delivery. However, it failed to find statistically significant milk microbial composition alterations between elective CS and vaginal delivery 3 months postpartum [106]. Analysis of colostrum and transitional milk, but not mature milk, revealed higher total bacterial concentrations in the CS group. It also revealed substantial differences in microbial composition, e.g., *Bifidobacterium* was more common in colostrum and transitional milk of vaginally delivering mothers, whereas *Streptococcus* spp. was more abundant in their colostrum. However, in comparison to other studies, in this study, no significant differences were observed, e.g., for *Enterococcus*, *Staphylococcus*, or *Lactobacillus* [107]. This shows that although many studies report differences in microbial composition, the specific microbial changes identified vary across studies, which could be due to, e.g., different sample collection times and geographical differences in delivery protocols. The fact that some articles combined elective and non-elective CS into a single group [107], whereas others did not [106], may also have influenced the results.

There are, however, also studies that failed to identify clear associations between delivery mode and human milk microbiota composition. Analyses using 16S rRNA sequencing have reported no significant differences in bacterial composition in transitional and mature milk based on delivery mode [108]. The authors of this study have even questioned the methodology of a different study that showed an effect of CS on microbiota composition, suggesting that the observed discrepancies might result from confounding factors (like body weight) rather than the impact of CS itself [108]. Similarly, another study reported no differences in microbiota diversity or richness in transitional and mature milk, but the analysis was limited by a very low sample size [109].

In addition, one study found a greater abundance of *Streptococcus mitis* in vaginally delivering mothers compared to emergency CS mothers (no specific sampling time). Human milk from mothers after CS also showed a trend toward higher bacterial richness than milk from mothers after vaginal delivery. It wasn’t statistically significant, though. Differences in early milk microbiota composition may reflect factors such as intrapartum antibiotic use and higher frequency of breastfeeding after vaginal delivery (CS is associated with breastfeeding difficulties after delivery), which can increase the presence of oral-origin bacteria in breast milk [110].

Overall, the negative effect of CS on milk microbiota, especially in colostrum, cannot be ruled out. Discrepancies between studies may also arise from variations in delivery practices and antibiotic regimens across countries or regions. More studies are required to fully assess the impact of CS on milk microbiota.

Regarding the mycobiota content of transient and mature human milk, in mothers of vaginally delivered neonates, it was more homogeneous, while more extreme values and higher averages of several fungi species were noted in mothers after CS. However, no statistically significant differences in overall fungal diversity and richness were observed [111].

Furthermore, substantial differences in milk viriome composition have been observed between CS and vaginal delivery [112]. Although the exact impact of milk viral components is still unknown, they may help shape a correct neonate and infant microbiome, especially bacteriophages. Changes in milk viriome composition could potentially pose a greater risk for infectious or autoimmune diseases, although beneficial effects are also possible [113]. Vaginal delivery was linked with a higher abundance of bacteriophages. Transient milk (collected 7–15 days postpartum) of term vaginal delivery mothers contained Podoviridae taxa predominantly, while mature milk (collected 45–90 days postpartum) contained predominantly Siphoviridae taxa. The group of term-CS mothers had a higher viral variety, but Myoviridae remained the most abundant in both transient and mature milk. The authors showed that the mode of delivery could affect the human milk virome, but noted that it cannot be attributed only to the delivery mode, as many factors could affect human milk virome composition, including lifestyle and gestational age [112].

There are also reported differences in certain colostrum human milk oligosaccharides (HMOs)—significant components of human milk that help shape correct neonate/infant intestinal microbiota [114]. Available data are, again, quite inconsistent. Many HMO types were found more abundant in the colostrum of mothers who underwent vaginal delivery than in the colostrum of mothers after CS [115]. However, a different study, which performed a similar analysis, reported that only non-fucosylated HMOs differed between CS and vaginal delivery mothers and were lower in the vaginal delivery group, not in the CS group [114]. Another, similar study, but performed 3/4 months postpartum, did not reveal any differences in terms of mode of delivery [116].

Based on the available data, it cannot be ruled out that CS is a risk factor for alterations in breast milk components that affect the neonatal/infant intestinal microbiota composition. However, certain studies have reported no significant, contradictory differences between delivery modes and microbiota or HMO composition [102,116]. These discrepancies may arise from multiple factors—including different sample timing [107], dietary patterns [100], geographical differences in milk microbiota composition [117], and grouping emergency and elective CS as a single CS category [107,112]. Additional factors related to different CS protocols across hospitals and countries—e.g., in antibiotic prophylaxis [110] or overall maternal care designed to reduce birth-related stress and support lactation [30,118]—may also contribute. Further studies that account for these variables are needed to establish and explain the exact impact of CS on milk microbiota composition.

### 3.7. miRNA

The importance of milk-derived microRNAs (miRNAs) has gained increasing attention in recent years. Human milk contains around 1400 different miRNAs [50]. Experimental and observational studies report that these milk molecules can cross the intestinal barrier and reach different organs, including the thymus and spleen [119]. They are believed to modulate infant immune function [120,121], metabolic pathways [122], and, e.g., neurogenesis [123,124].

Interestingly, the mode of delivery may also influence milk miRNA composition. In mature milk collected at six weeks postpartum, total miRNA abundance was lower in mothers after CS, and the levels of 22 individual miRNAs were significantly reduced relative to vaginal delivery. Functional analyses suggest that these miRNAs regulate pathways involved in satiety signaling (e.g., CCKR signaling) as well as mammary gland development and lactation, including FGF and EGF receptor signaling [53]. Conversely, another study reported higher levels of several miRNA families—let-7, 125, 30, 15, and 17—in the colostrum of women who delivered via CS compared to those with vaginal delivery [50]. Because these miRNAs are involved in immunomodulation and immune response execution, their altered expression may contribute to an increased risk of immune-related disorders later in life. The exact consequences of these changes remain unclear, highlighting the need for further research on this topic.

Collectively, these findings suggest that CS may disrupt miRNA profiles in both colostrum and mature milk, with potential consequences for neonatal and infant development. Notably, the specific miRNAs affected differ between colostrum and mature milk, and the direction of change is often opposite—upregulated in colostrum but downregulated in mature milk. Although these observations require further validation, they indicate that the relationship between delivery mode and milk miRNA content is dynamic and may vary across different stages of lactation.

### 3.8. Polyamines

Polyamines play important roles in gastrointestinal maturation and immune system development in the first years of life [125]. Among spermidine, spermine, and putrescine—the most common polyamines present in human milk—putrescine concentrations appear to correlate with delivery mode in certain countries. Higher putrescine concentrations have been reported in the mature milk of mothers who delivered via CS. However, the authors noted that geographical location had a far stronger influence on milk polyamine levels than delivery mode [126], likely reflecting differences in maternal dietary polyamine intake across populations [127]. Hypothetically, polyamine intake could differ slightly between mothers who undergo CS and those who deliver vaginally, which might explain the observed differences in milk putrescine levels.

## 4. Discussion

Based on the data mentioned above, CS is not only associated with the delayed initiation of lactation but may also affect breast milk composition, which is particularly visible in colostrum samples. These differences affect, e.g., nutrient content [54], mineral levels [46], immune-related molecules [51,81], hormones [88], antioxidant balance [48], microbiota [105], and miRNA profiles [50].

In terms of nutrient composition, CS appears to be associated with transient, early postpartum differences—such as slightly lower protein [54] or triglyceride [56] concentrations in colostrum. Although these changes may be less favorable for the newborn, they likely diminish as lactation progresses [57].

Regarding mineral content, the most consistent finding is elevated iodine concentrations in CS colostrum. This could be due to higher perioperative iodine exposure after CS. However, it remains unclear whether this short-term high iodine exposure has any clinically relevant negative effects on the neonate [46,68].

Immune and growth-factor composition also seems to be affected by delivery mode. CS was found to be associated with higher colostrum concentrations of, e.g., TGF-β1 and TGF-β2 [74], mucin 1 [75], IgA [74], and certain pro-inflammatory cytokines [81]. These molecules support the neonatal immune system development and protect against external pathogens. However, CS colostrum may also exhibit lower concentrations of antimicrobial β-defensins [87], as well as IGF-1 [79], which are also beneficial for newborns’ immunity.

In addition, hormonal differences in milk have been observed between CS and vaginal delivery, including lower β-endorphin [47] and disturbed melatonin levels [83,91] after CS. Although β-endorphin and melatonin are beneficial components of milk, there is insufficient evidence to determine the clinical relevance of their disturbed levels in the breast milk of mothers after CS.

Antioxidant and miRNA profiles are probably also affected by the mode of delivery. Colostrum of CS mothers showed higher oxidative stress markers and reduced total antioxidant capacity [94]. Certain colostrum miRNAs have been found elevated in CS mothers [50], while other miRNAs have been found at lower concentrations in mature milk [53]. Some of these miRNAs are involved in immunomodulation and immune responses. Their dysregulation in milk could increase the risk of developing immune system-related conditions later in life, e.g., allergies [50].

Finally, alterations in milk microbiota and HMOs have also been observed after CS, although many discrepancies exist—particularly in mature milk. Several studies note reduced microbial diversity [49], increased abundance of opportunistic or environmental potentially pathogenic bacteria, especially in colostrum [105]. Shifts in the milk virome [112] and mycobiome [111] after CS have also been noted. However, some studies did not find any significant associations between mode of delivery and microbiota composition, especially in mature milk, underscoring the role of confounding factors [108,109].

Overall, it is difficult to conclude whether CS negatively/positively impacts breast milk composition. Many observations indicate that CS may slightly reduce the beneficial effect of breastfeeding, e.g., slightly negatively affecting its nutrient [45,56] or microbiota composition [49,54,55,57]. However, some changes—particularly in immune-related factors—could be potentially advantageous for the neonate/infant after CS. Most of these observations appear to be transient and are primarily observed in colostrum, in early stages of lactation only [46]. Although long-term CS-related alterations in mature milk composition cannot be completely excluded [104], many reported differences—particularly in nutrient profiles and microbiota—are inconsistent and may reflect maternal age, lifestyle, or other factors that commonly correlate with CS rather than a direct effect of the procedure itself [65]. To date, no study has directly assessed whether these differences in milk composition have any significant clinical consequences, highlighting the need for further research on this topic. Nevertheless, based on the available literature, it is unlikely that these changes have a major impact on long-term health outcomes in the offspring.

This study also has several limitations. First, many studies were based on small sample sizes [46,102]. Limited statistical power may have contributed to inconsistent findings and prevented the detection of true differences. Some authors have noted in their articles that trends might reach significance in larger cohorts [55]. Second, differences in study design, including variation in sampling times, make comparisons across studies challenging, as human milk composition, especially in colostrum and transient milk, dynamically changes over time [128]. In addition, studies varied in the maternal age ranges considered, pre-pregnancy BMI, and whether only term deliveries or both preterm and term deliveries were included, as well as in the consideration of multiparity. While some studies adjusted for selected covariates [47,57], others did not [68], which may have influenced the reported results.

Another important limitation is the inconsistent classification of CS across studies. Some studies specifically analysed only elective CS [94], others distinguished between elective and emergency CS [104], while some combined both types into a single CS category [107]. Because emergency CS often involves labor-associated hormonal, inflammatory, and microbial exposures that more closely resemble those of vaginal delivery than elective CS, it should always be treated as a separate category. Further studies comparing milk composition between elective and emergency CS would provide greater insight into this topic.

Additionally, variability in clinical practices across countries and hospitals—including antibiotic prophylaxis [110], anaesthesia protocols [95], and postoperative care [30,118]—may contribute to the discrepancies observed among studies. Finally, as noted above, none of the studies included long-term follow-up of infant or neonatal outcomes, which limits the interpretation of the sustained clinical significance of the reported findings.

In summary, although the available studies, especially in colostrum, suggest that CS may alter breast milk composition, further research is needed to confirm and fully assess the independent effect of CS on human milk composition. Nevertheless, even if CS milk proves to be slightly less beneficial, breastfeeding should and will likely remain the preferred feeding option for neonates/infants born by CS, as these neonates/infants may have a greater need for breast milk-derived components to help mitigate the adverse effects of CS on gut microbiota development and immune system maturation [13,129].

## 5. Conclusions

**Figure 2:**
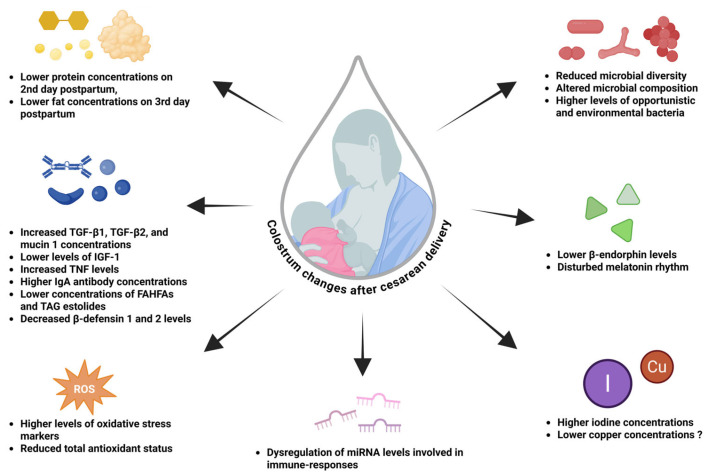
Colostrum composition changes following caesarean section. Created with BioRender.com.

Based on current evidence, the mode of delivery may affect breastmilk composition, especially colostrum, as illustrated and summarized in Figure 2. Although these effects appear to be largely transient, some studies suggest that certain alterations may persist into mature milk; however, findings at later lactation stages are often inconsistent and may be driven by methodological and external influences rather than true long-term effects of CS alone. Numerous factors contribute to variability in milk composition, including maternal body weight, preterm birth, postpartum care practices, ethnicity, antibiotic prophylaxis, parity, anaesthesia, and other perinatal variables. Further studies on this topic are required to fully assess the impact of CS on breast milk composition, ideally with larger sample sizes and rigorous addressing of the limitations identified in the presented literature.

## Data Availability

The datasets analysed for this study can be found in the MEDLINE database maintained by the United States National Library of Medicine (NLM) at the National Institutes of Health (https://pubmed.ncbi.nlm.nih.gov/, accessed 31 October 2025).

## Supplementary Materials

The following supporting information can be downloaded at: https://www.mdpi.com/article/10.3390/nu18020207/s1, Table S1: Articles Included in the Review.

## Abbreviation

The following abbreviation is used in this manuscript:

CS	Caesarean section
